# Facile synthesis of Mesoporouscobalt Hexacyanoferrate Nanocubes for High-Performance Supercapacitors

**DOI:** 10.3390/nano7080228

**Published:** 2017-08-21

**Authors:** Zhiyong Zhang, Jian-Gan Wang, Bingqing Wei

**Affiliations:** 1 State Key Laboratory of Solidification Processing, Center for Nano Energy Materials, School of Materials Science and Engineering, Northwestern Polytechnical University and Shaanxi Joint Lab of Graphene (NPU), Xi’an 710072, China; zyzhang@mail.nwpu.edu.cn; 2 Department of Mechanical Engineering, University of Delaware, Newark DE19716, USA

**Keywords:** prussian blue, electrode material, mesostructure, supercapacitor

## Abstract

Mesoporous cobalt hexacyanoferrate nanocubes (meso–CoHCF) were prepared for the first time through a facile sacrificial template method. The CoHCF mesostructures possess a high specific surface area of 548.5 m^2^·g^−1^ and a large amount of mesopores, which enable fast mass transport of electrolyte and abundant energy storage sites. When evaluated as supercapacitor materials, the meso–CoHCF materials exhibit a high specific capacitance of 285 F·g^−1^, good rate capability and long cycle life with capacitance retention of 92.9% after 3000 cycles in Na_2_SO_4_ aqueous electrolyte. The excellent electrochemical properties demonstrate the rational preparation of mesoporous prussian blue and its analogues for energy storage applications.

## 1. Introduction

Supercapacitors, also known as electrochemical capacitors, are considered one of the most promising energy storage devices, owing to their desirable properties of high power density, high energy density and excellent cycling stability [1]. Based on the charge-storage mechanism, supercapacitors can be generally classified into two types: electrical double-layer capacitors (EDLCs) and pseudocapacitors. The former stores charges through electrostatic adsorption/desorption at the electrode-electrolyte interface; while the energy of pseudocapacitors comes from rapid Faradaic reactions occurred at the surface of electrode materials [2]. For EDLCs, carbonaceous materials with large surface area are widely used, such as activated carbon [3], carbon nanotubes [4], carbon xerogel [5] and graphene [6]. The most widely investigated electrode materials of pseudocapacitors include conducting polymers and transition-metal oxides or hydroxides [7,8].

As a representative of the metal organic framework materials, prussian blue (PB) can be generally expressed as A*_x_*Fe[Fe(CN)_6_]*_y_*·*m*H_2_O (A, alkaline metal; 0 < *x* < 2, *y* < 1) [9,10]. In the framework of PB, Fe in low-spin and high-spin state bond with six C and six N atoms, respectively, forming face-centered-cube (FCC) crystal structure and resultant open channels [11,12,13]. Prussian blue analogues (PBAs), with similar crystal structures to PB, can be obtained by replacing part or all of the irons by other transition-metal elements (e.g., Co, Ni and Mn) [14,15]. As electrode materials, the open framework of PB or PBAs could provide large interstitial sites for insertion/extraction of alkali metals ions [16]. Besides, the PBAs possess the advantages of relatively higher specific capacitance, environmental friendliness and low cost, which make it promising in the field of energy storage [17]. However, the poor electrical conductivity and serious agglomeration of PBAs’ nanoparticles limit the effective utilization of their electrochemical performance [18]. To address this issue, two methods have been developed: (i) combining PBAs with conducting materials [19]; and (ii) increasing the reaction sites of PBAs by reducing the particle size with high specific surface area [20]. There have been a number of studies to synthesize PB/PBA and their composites for supercapacitors [21,22,23,24]. It is worth noting that mesopores are important for mass transport, which determines the accessing capability of electrolyte ions to the intrinsic micro-channels of PB/PBA [25]. However, the synthesis of mesoporous PB/PBAs has scarcely been reported.

In this work, we developed a facile and controllable sacrificial template route to prepare mesoporous cobalt hexacyanoferratenanocubes (meso–CoHCF). The as-prepared meso–CoHCF possesses a high specific surface area of 548.5 m^2^·g^−1^ and a large amount of mesopores centered at 4.6 nm. The meso–CoHCF electrodes exhibit a high specific capacitance of 285 F·g^−1^ at a scan rate of 2 mV·s^−1^ in neutral Na_2_SO_4_ electrolyte, which is much higher than that of the controlled CoHCF nanoparticles (CoHCF, 215 F·g^−1^).

## 2. Discussion and Results

### 2.1. Characterization of Meso–CoHCF

**Figure 1:**
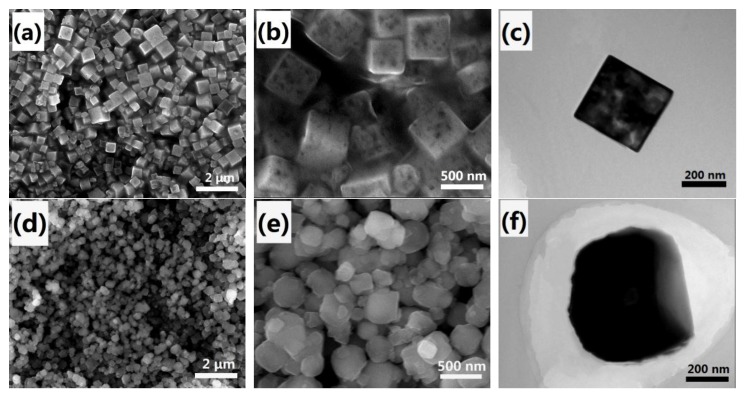
SEM and TEM images of meso–CoHCF nanocubes (**a**–**c**) and CoHCF nanoparticles (**d**–**f**).

Figure 1a–c exhibits the morphology of the meso–CoHCF sample. From the low magnification field-emission scanning electron microscopy (SEM) image (Figure 1a), the meso–CoHCF sample is composed of uniform nanocubes with sizes in the range of 300–500 nm. A closer observation from the high-resolution SEM image (Figure 1b) reveals the presence of mesopores on the exterior surface. The porous microstructure is verified by transmission electron microscopy (TEM) imaging, which shows the existence of numerous voids in the interior of the nanocubes. For comparison, CoHCF nanoparticles were synthesized by conventional chemical precipitation method. Figure 1d–f show the SEM and TEM images of the CoHCF nanoparticles. It is observed that the irregular CoHCF nanoparticles are of solid morphology.

**Figure 2:**
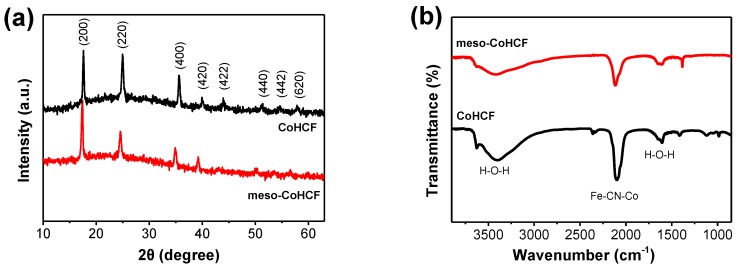
(**a**) XRD patterns and (**b**) FTIR spectraof meso–CoHCF and CoHCF.

The phase structure of the products is investigated by X-ray diffraction (XRD). As shown in Figure 2a, the meso–CoHCF and CoHCF samples show identical diffraction pattern, in which the diffraction peaks can be well indexed to the face-centered cubic phase of Co_3_[Fe(CN)_6_]_2_·*x*H_2_O [26]. No other diffraction peaks are noted, indicating a high purity of the products. To determine the chemical structure of CoHCF, Fourier-transform infrared (FTIR) spectra were collected. As shown in Figure 2b, the strong absorption band at 2104 cm^−1^ is characteristic of PB structure, which can be assigned to the stretching vibration of Fe–CN–Co bonds. The absorption peaks at 3407 and 1620 cm^−1^ can be attributed to the stretching and bending modes of the H−O−H, respectively, indicating the existence of the interstitial water in the crystal lattice of the sample [27].

**Figure 3:**
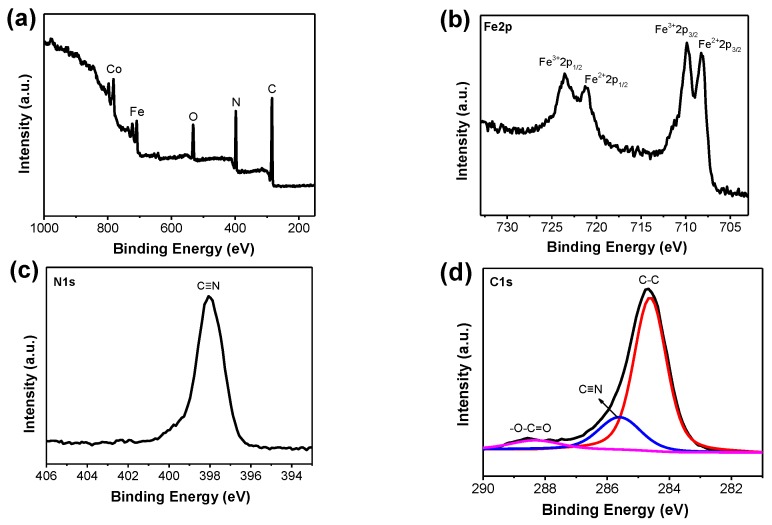
XPS spectra of meso–CoHCF: (**a**) full scan; (**b**) Fe2p; (**c**) N1s and (**d**) C1s.

The elemental components and their chemical states of the meso–CoHCF were examined by X-ray photoelectron spectroscopy (XPS) measurements. As shown in Figure 3a, the full scan spectrum shows the existence of Co, Fe, O, N and C elements in the sample. Two pairs of subpeaks are present in the high-resolution spectra of Fe2p (Figure 3b). The binding energies at 723.5 and 710.0 eV can be assigned to the Fe^3+^ in [Fe(CN)_6_]^3+^, while the peaks at 721.1 and 708.2 eV belong to the Fe^2+^ in [Fe(CN)_6_]^4+^ [27,28]. The spectrum of N1 s (Figure 3c) displays a distinct peak located at 397.9 eV, corresponding to the C≡N in [Fe(CN)_6_] groups [29]. The C 1s spectrum (Figure 3d) can be fitted by three component curves centered at 288.3, 285.6 and 284.6 eV, representing O–C=O, C≡N and C–C bonds, respectively.

**Figure 4:**
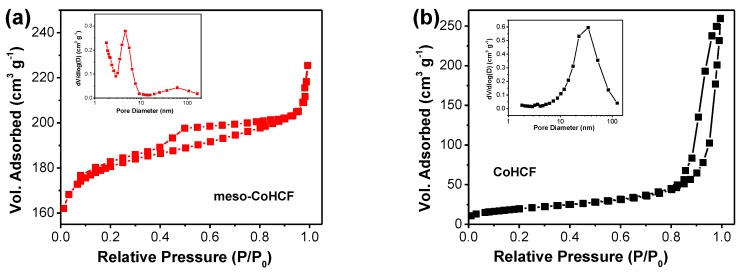
N_2_ adsorption/desorption isotherm and pore size distribution of (**a**) meso–CoHCF and (**b**) CoHCF.

The porous characteristics of the samples were investigated by N_2_ adsorption-desorption measurements. Figure 4a,b exhibits the resulting isotherms of meso–CoHCF and CoHCF. The meso–CoHCF possesses a large hysteresis loop in the middle P/P_0_ region, indicating the presence of uniform mesopores. The corresponding Barrett–Joyner–Halenda (BJH) pore-size distribution plots are displayed as inserts in Figure 4. It is noted that the meso–CoHCF sample has uniform mesopore size centered at 4.6 nm. In sharp contrast, the controlled CoHCF sample shows irregular large pores in the range of 10–100 nm, which come from the agglomeration of the nanoparticles. More remarkably, the Brunauer-Emmett-Teller (BET) specific surface area of the meso–CoHCF is as large as 548.5 m^2^·g^−1^, which is much higher than that of the CoHCF (70.0 m^2^·g^−1^). The high surface area along with the uniform mesoporous structure of the meso–CoHCF provide more accessible electro-active sites for charge storage and allow for easy transport of electrolyte ions.

### 2.2. Electrochemical Performance

**Figure 5:**
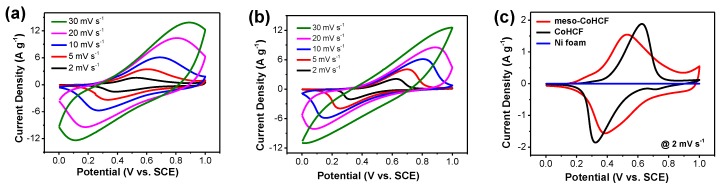
CV curves of (**a**) meso–CoHCF and (**b**) CoHCF electrodes and (**c**) their comparison at 2 mV·s^−1^.

The electrochemical properties of the meso–CoHCF electrode were evaluated by cyclic voltammetry (CV) and galvanostatic charge/discharge techniques in 0.5 M Na_2_SO_4_ aqueous electrolyte. Figure 5 shows the CV curves of the meso–CoHCF and CoHCF electrodes at various scan rates. Both electrodes exhibit typical pseudocapacitive behavior, which features a pair of redox peaks associated with transition between Fe^2+^ and Fe^3+^ in CoHCF [30]. The symmetric peak shape indicates good reaction reversibility. Compared to the CoHCF electrode (Figure 5b), the CV shape of the meso–CoHCF electrode is well maintained as the scan rate increases to 30 mV·s^−1^, indicating better rate capability of the meso–CoHCF electrode with small polarization. The larger current density of the meso−CoHCF electrode at each scan rate manifests a higher specific capacitance. In addition, it is interesting to note that the high-specific-surface-area meso–CoHCF electrode exhibits an approximately rectangular CV shape in the high potential region of 0.8–1.0 V, as shown in Figure 5c, which is totally different from the CoHCF electrode, demonstrating that there are some electrochemical double layer (EDL) capacitance contributing to the overall capacitance. More importantly, the specific capacitance of the meso–CoHCF electrode is as high as 285 F·g^−1^ at 2 mV·s^−1^, which is much higher than that of the controlled electrode (215 F·g^−1^). Moreover, the meso–CoHCF electrode exhibit better capacitance performance (e.g., 272 F·g^−1^ at 5 mV·s^−1^) than the reported manganese hexacyanoferrat/manganese dioxide (MnHCF/MnO_2_) composites electrode (225.6 F·g^−1^ at 5 mV·s^−1^) [21], CoHCF nanoparticles electrode (250 F·g^−1^ at 5 mV·s^−1^ ) [24], meso–NiHCF (184 F·g^−1^ at 5 mV·s^−1^) and meso–CuHCF (243 F·g^−1^ at 5 mV·s^−1^) [23]. 

**Figure 6:**
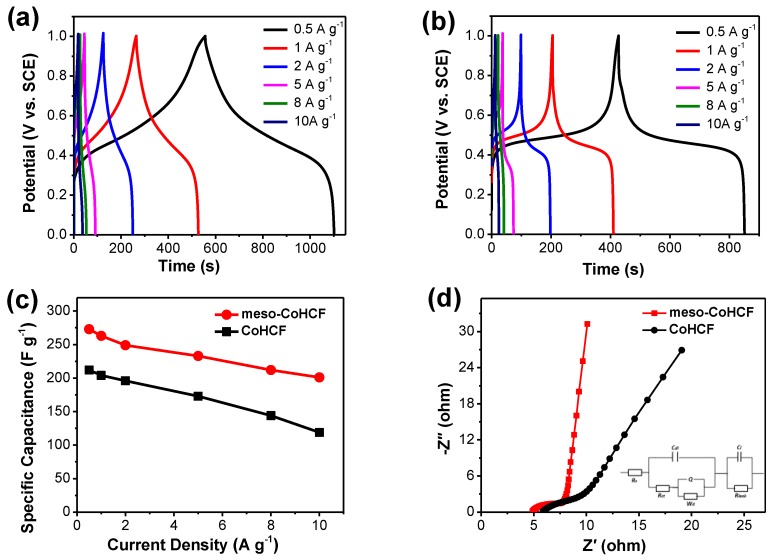
(**a**) Charge/discharge curves of meso–CoHCF electrode and (**b**) CoHCF electrode; and (**c**) their corresponding specific capacitance and (**d**) Nyquist plots.

Figure 6a,b shows the galvanostatic charge/discharge curves of the meso–CoHCF and CoHCF electrodes at different current densities from 0.5 to 10 A·g^−1^. Both curves exhibit a long charge/discharge voltage plateau at 0.3–0.6 V, which is consistent with the redox peaks in CV curves. The symmetric charge/discharge profiles indicate a high Coulombic efficiency. Figure 6c plots the specific capacitance of the meso–CoHCF and CoHCF electrodes at various current densities. The specific capacitance of both electrodes decreases with the increase of the current density due to the reduced diffusion time at a high current rate [31,32]. It is noted that the meso–CoHCF electrode can deliver a highspecific capacitance of 273 F·g^−1^ at 0.5 A·g^−1^, and sustains an excellent capacitance retention of 73.6% even at 10 A·g^−1^. However, the controlled CoHCF electrode can only deliver specific capacitance of 212 and 119 F·g^−1^ at 0.5 and 10 A·g^−1^, respectively. The higher specific capacitance along with the better rate capability of the meso–CoHCF electrode can be attributed to the unique porous structure: (i) the higher BET specific surface area provides more accessible electro-active sites for charge storage; and (ii) the uniform mesopores facilitate fast mass transport of electrolyte ions throughout the electrode for better reaction kinetics.

To further understand the electrochemical behavior of the electrodes, electrochemical impedance spectrum (EIS) measurements were performed and the resulting Nyquist plots are shown in Figure 6d. The EIS data can be fitted using an equivalent circuit model (insert). It can be observed that the EIS curves are composed of a semicircle in the high frequency region and a sloped line in the low frequency region. The first x-intercept on the real axis (*Z*’) represents the bulk resistance of the electrode (R_s_), and the diameter of the semicircle corresponds to the charge-transfer resistance (R_ct_) [33]. Notably, both R_s_ (4.9 Ω) and R_ct_ (3.2 Ω) of the meso–CoHCF electrode are much smaller than that of the CoHCF electrode (i.e., 6.1 and 4.3 Ω). The porous structure of the meso–CoHCF electrode provides more accessible sites for the charges accumulated at the surface, thereby resulting in a higher double layer capacitance (C_dl_) of around 85 μF than that of the CoHCF electrode (16 μF) [4]. In addition, the sloped line is associated with the electrolyte diffusion/transport into the porous electrode [32,33]. Clearly, the meso–CoHCF electrode possesses much more vertical shape, indicating lower diffusion resistance. It is anticipated that the lower internal and diffusion resistance of the meso–CoHCF electrode renders fast reaction kinetics for better power delivery.

**Figure 7:**
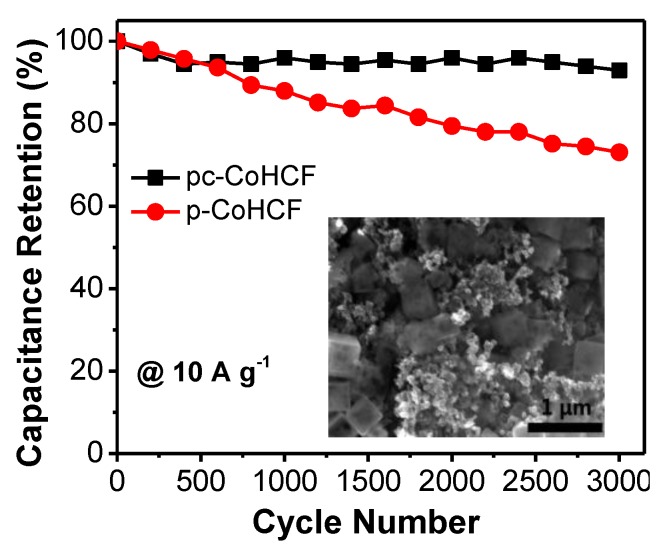
Cycle stability of meso–CoHCF and CoHCF electrodes. Inset shows the morphology of the meso–CoHCF electrode after cycling test.

Good cycling stability is an important criterion for supercapacitors in practical applications. Figure 7 shows the cycling performance of the meso–CoHCF and CoHCF electrodes at a large current density of 10 A·g^−1^. The meso–CoHCF electrode retains 92.9% of the initial capacitance after 3000 cycles, revealing long-term cycling stability. By contrast, the CoHCF electrode shows a serious capacitance degradation of 26.9% over the 3000-cycle test. The better cycling stability of the meso–CoHCF electrode may benefit from the voidspace in the interior of the nanocubes, which can accommodate the possible volume expansion and contraction during the long-term operation. To confirm it, the morphology of the meso–CoHCF electrode after cycling test is characterized. As shown in the inset of Figure 7, the nanocubes are well maintained after cycling, indicating the robust structure of the meso–CoHCF.

## 3. Materials and Methods 

Materials Synthesis: All chemical reagents were analytical grade and purchased from Sinopharm Chemical Reagent Co., Ltd. (Shanghai, China). In a typical synthesis procedure of the meso–CoHCF, 0.4 g of polyvinylpyrrolidone (PVP) and 100 mg of MnSO_4_H_2_O were dissolved in 10 mL ethanol and 10 mL H_2_O, respectively, under stirring for 30 min to form a homogeneous solution. Next, 10 mL of 0.03 M potassium ferricyanide (K_3_Fe(CN)_6_) was added in 3 min and kept under stirring overnight to form MnHCF template. Then, 10 mL of 0.045 M Co(NO_3_)_2_6H_2_O was mixed with the above suspension and let stand for 5 h at 50 °C. Finally, the meso–CoHCF products were obtained after filtration (the pore diameter of filter paper was ~0.45 μm) and washing with deionized water and finally drying at 80 °C for 12 h. For comparison, CoHCF nanoparticles were prepared by directly mixing 20 mL of 15 mM Co(NO_3_)_2_ with 20 mL 10 mM K_3_Fe(CN)_6_ under stirring for 3 h. The precipitates were filtered, rinsed and dried using a similar process. 

Materials Characterization: The crystalline structures of the as-prepared products were characterized by X-ray powder diffraction (XRD, X’Pert Pro MPD, Philips, Almelo, The Netherlands). The Fourier-transform infrared (FT-IR, Nicolet iS50, Thermo Fisher Scientific, Waltham, MA, USA) spectra were collected in the region from 800 to 4000 cm^−1^ to detect the chemical components of the samples. X-ray photoelectron spectroscopy (XPS, ESCALAB 250Xi, Thermo Scientific, Waltham, MA, USA) measurements were applied to investigate the elemental composition and surface chemistry. The morphology of the samples was observed with field emission scanning electron microscopy (FE-SEM, FEI NanoSEM 450, FEI, Portland, OR, USA) and transmission electron microscopy (TEM, FEI Tecnai F30G2, FEI, Portland, OR, USA). The porous characteristics of the products was measured by N_2_adsorption-desorption isotherms (ASAP 2020, Mike, Norcross, GA, USA). The specific surface area was calculated using the BET method while the pore size distribution was determined by BJH model.

Electrochemical Measurements:The electrochemical tests of meso–CoHCF electrodes were performed on an electrochemical workstation (Solartron 1260 + 1287, Bognor Regis, West Sussex, UK) with a three-electrode configuration system. For the preparation of the working electrode, the active materials, carbon black and polytetrafluoroethylene (PTFE) were mixed with a mass ratio of 7:2:1 and stirred for 2 h. The working electrode was yielded after uniformly painting the above slurry on the nickel foam (1 × 2 cm) and drying at 90 °C for 12 h in an oven. Platinum foil and saturated calomel electrode (SCE) served as counter electrode and reference electrode, respectively, and a 0.5 M Na_2_SO_4_ aqueous solution was employed as electrolyte. The mass loading of active materials on the working electrode was around 2.0 mg·cm^−2^. The cyclic voltammetry (CV) and galvanostatic charge-discharge techniques were employed within a potential window ranging from 0.0 to 1.0 V. to evaluate the electrochemical performance of the working electrodes [34]. The electrochemical impedance spectroscopy (EIS) measurements were conducted in the frequency range of 10 kHz to 0.01 Hz.

## 4. Conclusions 

A facile chemical-processing method has been developed in our work to prepare the mesoporous CoHCF nanocubes for supercapcitors. The meso–CoHCF with size between 300 and 500 nm show a high specific surface area of 548.5 m^2^·g^−1^ and sufficient meso–channels, which not only offers abundant electro-active sites but also facilitates the mass transport of electrolyte. The meso–CoHCF electrodes exhibit a high specific capacitance of 285 F·g^−1^ at a scan rate of 2 mV·s^−1^ and excellent rate capability in Na_2_SO_4_ aqueous electrolyte. In addition, the capacitance retention is as high as 92.9% after 3000 cycles at a large current density of 10 A·g^−1^. The high specific capacitance, good rate capability and long cycle life makethe meso–CoHCFpromising as supercapacitor electrode materials for practical applications.

## References

[1] Wang J., Kang F., Wei B. (2015). Engineering of MnO_2_-based nanocomposites for high-performance supercapacitors. Prog. Mater. Sci..

[2] Li H., Yu M., Wang F., Liu P., Liang Y., Xiao J., Wang C., Tong Y., Yang G. (2013). Amorphous nickel hydroxide nanospheres with ultrahigh capacitance and energy density as electrochemical pseudocapacitor materials. Nat. Commun..

[3] Li B., Dai F., Xiao Q., Yang L., Shen J., Zhang C., Cai M. (2016). Nitrogen-doped activated carbon for a high energy hybrid supercapacitor. Energ. Environ. Sci..

[4] Masarapu C., Zeng H., Wei B. (2009). Effect of temperature on the capacitance of carbon nanotube supercapacitors. ACS Nano.

[5] Wang J.-G., Zhang C., Jin D., Xie K., Wei B. (2015). Synthesis of ultralong MnO/C coaxial nanowires as freestanding anodes for high-performance lithium ion batteries. J. Mater. Chem. A.

[6] Peng Z., Lin J., Ye R., Samuel E., Tour J. (2015). Flexible and stackable laser-induced graphene supercapacitors. ACS Appl. Mater. Interfaces.

[7] Wang J.-G., Jin D., Zhou R., Shen C., Xie K., Wei B. (2016). One-step synthesis of NiCo_2_S_4_ ultrathin nanosheets on conductive substrates as advanced electrodes for high-efficient energy storage. J. Power Sources.

[8] Chen H., Hu L., Chen M., Yan Y., Wu L. (2014). Nickel-cobalt layered double hydroxide nanosheets for high-performance supercapacitor electrode materials. Adv. Funct. Mater..

[9] Yue Y., Binder A., Guo B., Zhang Z., Qiao Z., Tian C., Dai S. (2014). Mesoporous prussian blue analogues: Template-free synthesis and sodium-ion battery applications. Angew. Chem. Int. Edit..

[10] Wang J.-G., Jin D., Liu H., Zhang C., Zhou R., Shen C., Xie K., Wei B. (2016). All-manganese-based Li-ion batteries with high rate capability and ultralong cycle life. Nano Energy.

[11] Liu S., Pan G., Li G., Gao X. (2015). Copper hexacyanoferrate nanoparticles as cathode material for aqueous Al-ion batteries. J. Mater. Chem. A.

[12] Su D., McDonagh A., Qiao S., Wang G. (2017). High-capacity aqueous potassium-ion batteries for large-scale energy storage. Adv. Mater..

[13] Kong B., Selomulya C., Zheng G., Zhao D. (2015). New faces of porous prussian blue: Interfacial assembly of integrated hetero-structures for sensing applications. Chem. Soc. Rev..

[14] Shiba F., Fujishiro R., Kojima T., Okawa Y. (2012). Preparation of monodisperse cobalt(II) hexacyanoferrate(III) nanoparticles using cobalt ions released from a citrate complex. J. Phys. Chem. C.

[15] Subramani K., Jeyakumar D., Sathish M. (2014). Manganese hexacyanoferrate derived Mn_3_O_4_ nanocubes-reduced graphene oxide nanocomposites and their charge storage characteristics in supercapacitors. Phys. Chem. Chem. Phys..

[16] Mizuno Y., Okubo M., Hosono E., Kudo T., Oh-ishi K., Okazawa A., Kojima N., Kurono R., Nishimura S., Yamada A. (2013). Electrochemical Mg^2+^ intercalation into a bimetallic CuFe prussian blue analog in aqueous electrolytes. J. Mater. Chem. A.

[17] You Y., Wu X., Yin Y., Guo Y. (2014). High-quality prussian blue crystals as superior cathode materials for room-temperature sodium-ion batteries. Energy Rnviron. Sci..

[18] Zou Y., Wang Q., Xiang C., She Z., Chu H., Qiu S., Xu F., Liu S., Tang C., Sun L. (2016). One-pot synthesis of ternary polypyrrole–prussian-blue–graphene-oxide hybrid composite as electrode material for high-performance supercapacitors. Electrochim. Acta.

[19] Wang J., Zhang Z., Liu X., Wei B. (2017). Facile synthesis of cobalt hexacyanoferrate/graphene nanocomposites for high-performance supercapacitor. Electrochim. Acta.

[20] Moo Lee K., Tanaka H., Ho Kim K., Kawamura M., Abe Y., Kawamoto T. (2013). Improvement of redox reactions by miniaturizing nanoparticles of zinc prussian blue analog. Appl.Phys.Lett..

[21] Wang Y., Zhong H., Hu L., Yan N., Hu H., Chen Q. (2013). Manganese hexacyanoferrate/MnO_2_ composite nanostructures as a cathode material for supercapacitors. J. Mater. Chem. A.

[22] Wang Y., Chen Q. (2014). Dual-Layer-Structured nickel hexacyanoferrate/MnO_2_ composite as a high-energy supercapacitive material based on the complementarity and interlayer concentration enhancement effect. ACS Appl. Mater. Interfaces.

[23] Yue Y., Zhang Z., Binder A. (2015). Hierarchically superstructured prussian blue analogues: Spontaneous assembly synthesis and applications as pseudocapacitive materials. ChemSusChem.

[24] Zhao F., Wang Y., Xu X., Liu Y., Song R., Lu G., Li Y. (2014). Cobalt hexacyanoferrate nanoparticles as a high-rate and ultra-stable supercapacitor electrode material. ACS Appl. Mater. Interfaces..

[25] Yue Y., Fulvio P.F., Dai S. (2015). Hierarchical Metal–Organic Framework Hybrids: Perturbation-Assisted Nanofusion Synthesis. Acc. Chem. Res..

[26] Lu K., Song B., Gao X., Dai H., Zhang J., Ma H. (2016). High-energy cobalt hexacyanoferrate and carbon micro-spheres aqueous sodium-ion capacitors. J. Power Sources.

[27] Wang J.-G., Zhang Z., Zhang X., Yin X., Li X., Liu X., Kang F., Wei B. (2017). Cation exchange formation of prussian blue analogue submicroboxes for high-performance Na-ion hybrid supercapacitors. Nano Energy.

[28] Wu X., Luo Y., Sun M., Qian J., Cao Y., Ai X., Yang H. (2015). Low-defect prussian blue nanocubes as high capacity and long life cathodes for aqueous Na-ion batteries. Nano Energy.

[29] Luo X., Pan J., Pan K., Yu Y., Zhong A., Wei S., Li J., Shi J., Li X. (2015). An electrochemical sensor for hydrazine and nitrite based on graphene–cobalt hexacyanoferrate nanocomposite: Toward environment and food detection. J. Electroanal. Chem..

[30] Luo M., Dou Y., Kang H., Ma Y., Ding X., Liang B., Ma B., Li L. (2015). A novel interlocked prussian blue/reduced graphene oxide nanocomposites as high-performance supercapacitor electrodes. J. Solid State Electrochem..

[31] Wang J., Yang Y., Huang Z., Kang F. (2011). Coaxial carbon nanofibers/MnO_2_ nanocomposites as freestanding electrodes for high-performance electrochemical capacitors. Electrochim. Acta.

[32] Wang J., Yang Y., Huang Z. (2013). A high-performance asymmetric supercapacitor based on carbon and carbon–MnO_2_ nanofiber electrodes. Carbon.

[33] Wang J., Jin D., Zhou R., Li X., Liu X., Shen C., Xie K., Li B., Kang F., Wei B. (2016). Highly flexible graphene/Mn_3_O_4_ nanocomposite membrane as advanced anodes for Li-ion batteries. ACS Nano.

[34] Younis A., Chu D., Li S. (2015). Ethanol-directed morphological evolution of hierarchical CeO*_x_* architectures as advanced electrochemical capacitors. J. Mater. Chem. A.

